# Supplementary material for: EGFL7 enhances surface expression of integrin α5β1 to promote angiogenesis in malignant brain tumors
Source: EMBO Mol Med. 2018 Aug 2;10(9):e8420. doi: 10.15252/emmm.201708420 (PMC6127886; doi:10.15252/emmm.201708420)
Supplement: Supplementary file 1 — Appendix [file EMMM-10-e8420-s001.pdf]

EGFL7 enhances surface expression of integrin $\alpha_5\beta_1$ to promote angiogenesis in malignant brain tumors

Nevenka Dudvarski Stanković*, Frank Bicker*, Stefanie Keller*, David T. W. Jones, Patrick N. Harter, Arne Kienzle, Clarissa Gillmann, Philipp Arnold, Anna Golebiewska, Olivier Keunen, Alf Giese, Andreas von Deimling, Tobias Bäuerle, Simone P. Niclou, Michel Mittelbronn, Weilan Ye, Stefan Pfister & Mirko H.H. Schmidt

Appendix

Table of Content **Page**

Figure S1 1

Figure S2 3

Figure S3 5

Figure S4 7

Figure S5 8

Figure S6 10

Table S1 12

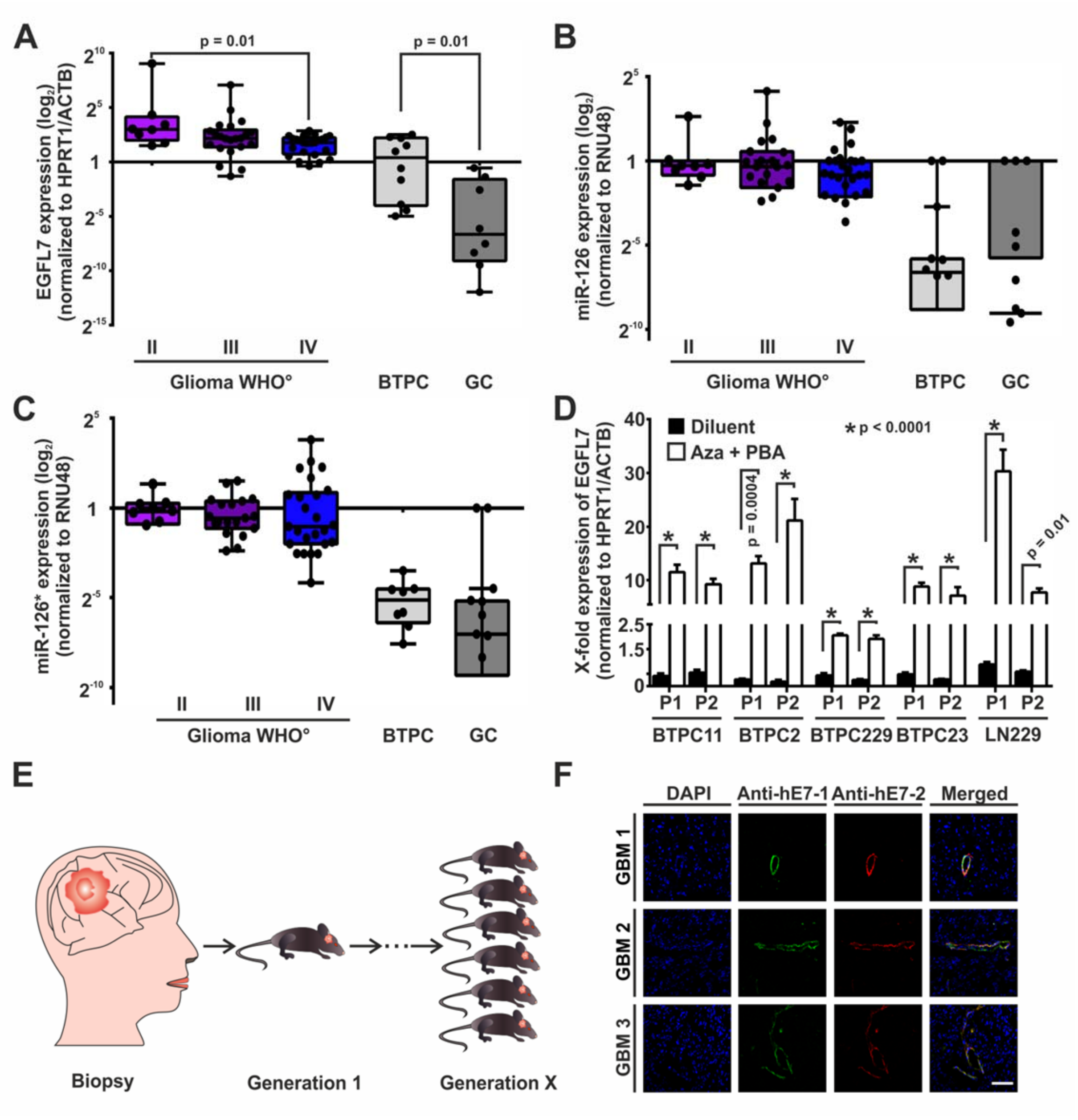

Figure S1. EGFL7 and miR-126 expression in glioma

(**A**) EGFL7 expression was analyzed by qRT-PCR in glioma specimens (n = 8 astrocytoma WHO° grade II, n = 22 astrocytoma WHO° grade III, n = 24 glioma WHO° grade IV), stem-like brain tumor-propagating cells (BTPC; n = 10), and glioma-derived cells (GC; n = 8). Expression was detected in tumor biopsies, but little to no expression was measured in BTPCs and GCs (one-way ANOVA). (**B**) miR-126 and (**C**) miR-126* expression analysis by Taqman in the same samples revealed no significant differences between the investigated groups. (**D**) Treatment

of BTPCs and GC LN229 with methyltransferase inhibitor Aza (5-Aza-dC) and histone deacetylase inhibitor (PBA) promoted EGFL7 expression in all samples as measured by qRT-PCR (P1 - first passage, P2 - second passage; n = 3; Mann-Whitney U-test). (**E**) Schematic presentation of the Patient-derived xenograft (PDX) model. Human glioma biopsies were serially implanted into mice for various generations without cultivation *in vitro*. (**F**) Immunohistochemical co-staining of glioma specimens using two independent anti-human EGFL7 antibodies confirmed mutual target specificity. Data presented as mean ± SEM. Scale bar represents 200 µm.

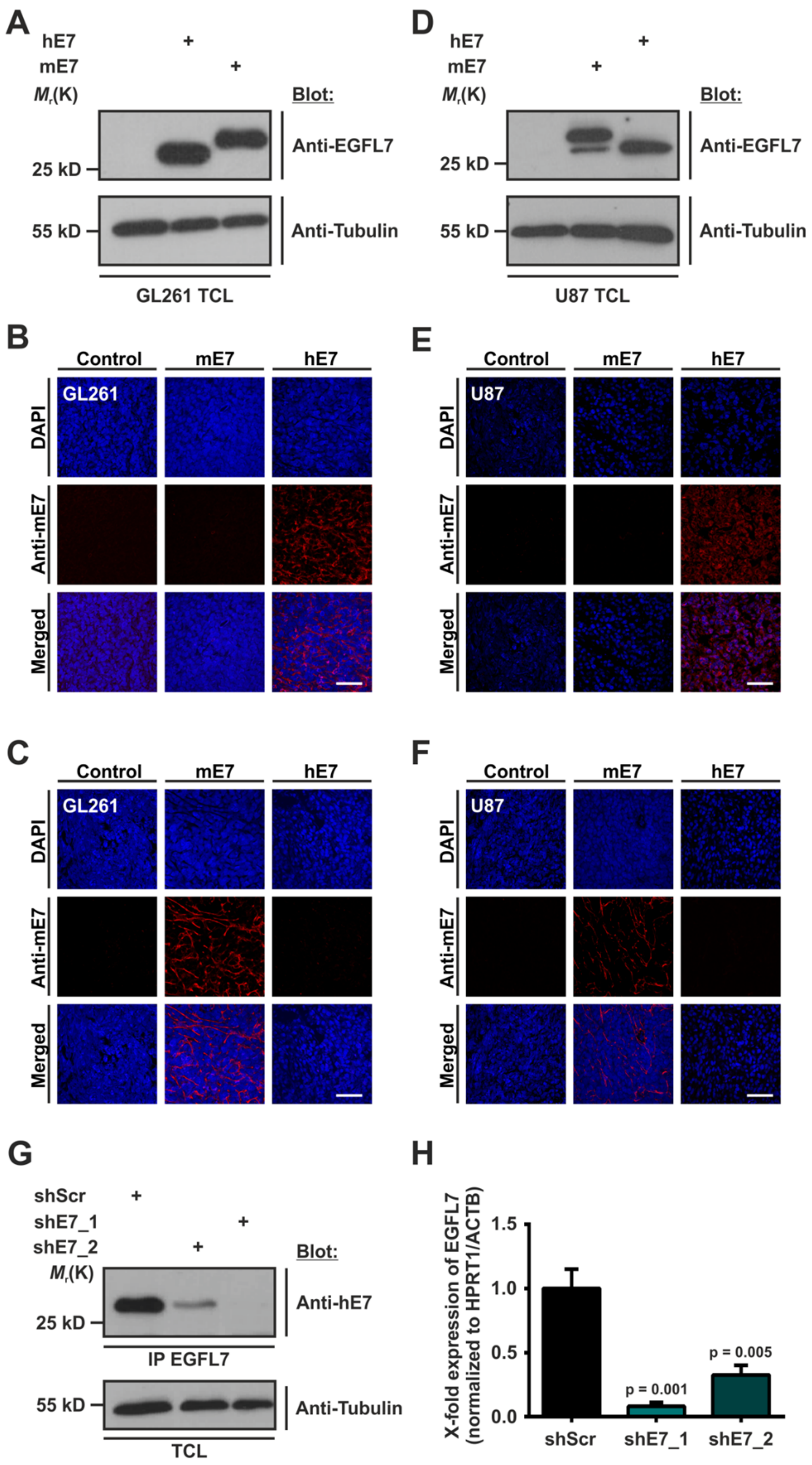
A
hE7
mE7
M_r(K)
Blot:
Anti-EGFL7
25 kD
55 kD
Anti-Tubulin
GL261 TCL
D
U87 TCL
B
Control
mE7
hE7
GL261
DAPI
Anti-mE7
Merged
E
U87
C
F
G
shScr
shE7_1
shE7_2
Anti-hE7
IP EGFL7
TCL
H
X-fold expression of EGFL7 (normalized to HPRT1/ACTB)
p = 0.001
p = 0.005
1.5
1.0
0.5
0.0

Figure S2. Experimental glioma model expression controls

(**A**) Immunoblotting confirmed successful expression of human (hE7) and mouse (mE7) EGFL7 in transduced mouse GL261 cells. (**B+C**) Verification of hE7 or mE7 expression in GL261 tumors by immunohistochemistry. Cell nuclei were counterstained with DAPI staining. (**D-F**) Likewise, expression of hE7 and mE7 was confirmed in human U87 cells. Scale bars represent 60 µm. (**G**) Immunoprecipitation of hE7 followed by western blot analysis or (**H**) qRT-PCR showed >90% and >70% decreased expression of EGFL7 after treatment with shRNA shE7_1 or shE7_2 (n = 3; one-way ANOVA). All data are presented as mean ± SEM.

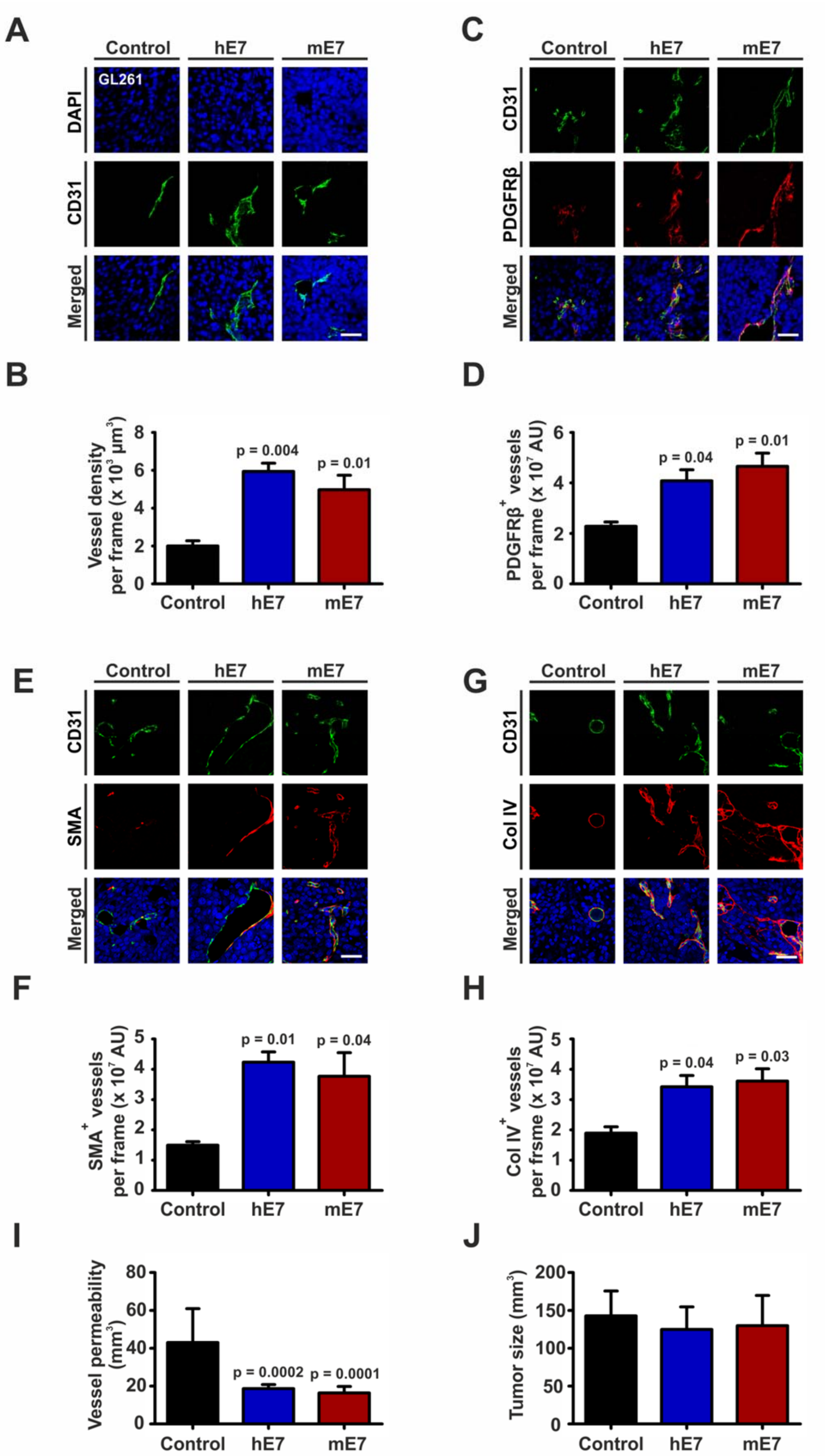
A
Control
hE7
mE7
GL261
DAPI
CD31
Merged
B
Vessel density per frame (x 10^3 µm^3)
p = 0.004
p = 0.01
Control
hE7
mE7
C
Control
hE7
mE7
CD31
PDGFRβ
Merged
D
PDGFRβ$^+$ vessels per frame (x 10^7 AU)
p = 0.04
p = 0.01
Control
hE7
mE7
E
Control
hE7
mE7
CD31
SMA
Merged
F
SMA$^+$ vessels per frame (x 10^7 AU)
p = 0.01
p = 0.04
Control
hE7
mE7
G
Control
hE7
mE7
CD31
Col IV
Merged
H
Col IV$^+$ vessels per frsme (x 10^7 AU)
p = 0.04
p = 0.03
Control
hE7
mE7
I
Vessel permeability (mm^3)
p = 0.0002
p = 0.0001
Control
hE7
mE7
J
Tumor size (mm^3)
Control
hE7
mE7

Figure S3. EGFL7 promoted density and maturation state of glioma vessels

To assess tumor vascularization, mice were intrastriatally implanted with human EGFL7 (hE7) or murine EGFL7 (mE7) expressing GL261 cells. Mice were sacrificed upon showing first symptoms of disease and brains were analyzed by magnetic resonance imaging (MRI). Resulting brain tumor sections were analyzed for blood vessel density and maturation state by immunohistochemistry. (**A+B**) CD31 staining for endothelial cells revealed increased tumor vascularization in mice bearing hE7- and mE7-positive tumors (n = 3; one-way ANOVA). Enhanced maturation of glioma vessels in the presence of E7 was verified by increased co-localization of (**C+D**) PDGFRβ (pericytes), (**E+F**) SMA (smooth muscle cells), or (**G+H**) Col IV (basement membrane) with CD31 (n = 3*;* one-way ANOVA; quantifications normalized to CD31). (**I**) T1-weighted MRI images showed decreased vessel permeability as measured by Gadovist extravasation in tumors expressing hE7 or mE7 (n = 7; one-way ANOVA*)*. (**J**) T2-weighted MRI analysis confirmed that all mice developed tumors of similar size (n = 7; n.s. – not significant; one-way ANOVA). Data presented as mean ± SEM, AU-arbitrary units. Scale bars represent 60 µm.

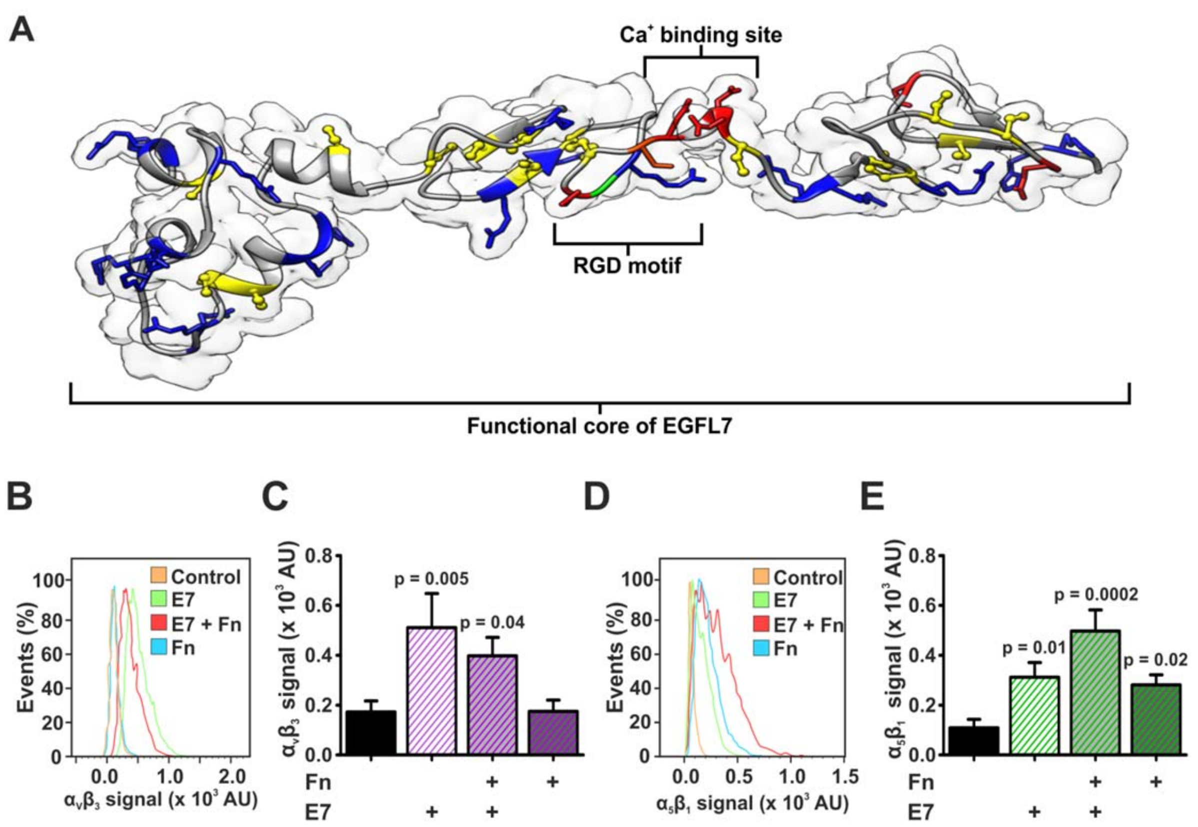

Figure S4. EGFL7 increased surface expression of integrins $\alpha_5\beta_1$ and $\alpha_V\beta_3$

(**A**) Model of the EGFL7 crystal structure with the RGD integrin-binding motif exposed in the elongated form of EGFL7. (**B+C**) FACS analysis revealed increased surface expression of $\alpha_V\beta_3$ upon application of EGFL7 (E7) but not fibronectin (Fn) alone (n = 3; one-way ANOVA). (**D+E**) Surface expression of $\alpha_5\beta_1$ was increased by E7 or Fn and further enhanced upon combination of both (n = 3; one-way ANOVA).

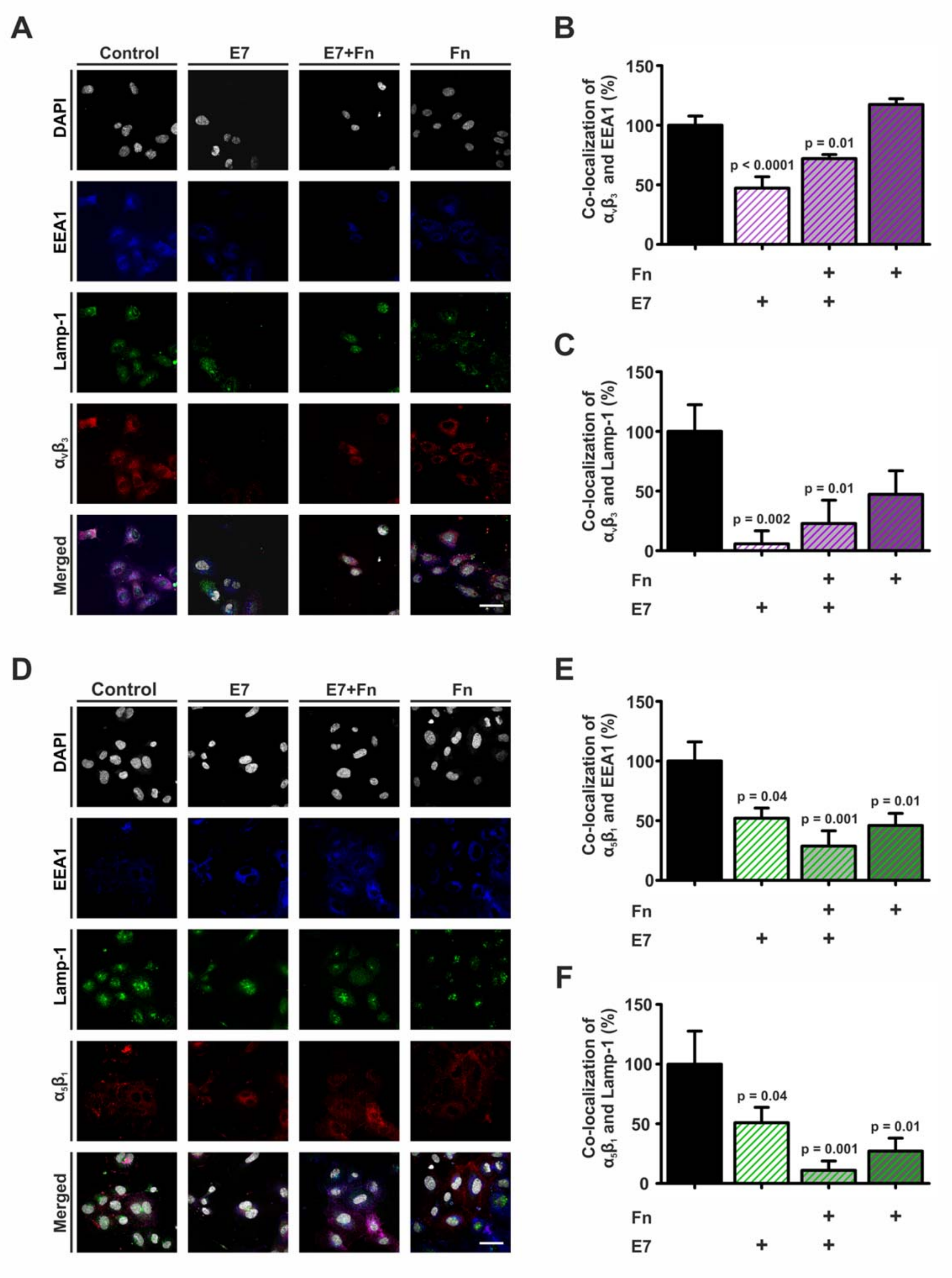
A
Control
E7
E7+Fn
Fn
DAPI
EEA1
Lamp-1
$\alpha_v\beta_3$
Merged
B
Co-localization of $\alpha_v\beta_3$ and EEA1 (%)
p < 0.0001
p = 0.01
Fn
E7
C
Co-localization of $\alpha_v\beta_3$ and Lamp-1 (%)
p = 0.002
p = 0.01
D
$\alpha_5\beta_1$
E
Co-localization of $\alpha_5\beta_1$ and EEA1 (%)
p = 0.04
p = 0.001
p = 0.01
F
Co-localization of $\alpha_5\beta_1$ and Lamp-1 (%)

Figure S5. EGFL7 reduced intracellular localization of integrins $\alpha_5\beta_1$ and $\alpha_V\beta_3$

(**A-C**) Colocalization studies of integrin $\alpha_V\beta_3$ in primary endothelial cells (HUVEC) along with early endosome antigen 1 (EEA1) and lysosomal-associated membrane protein 1 (Lamp-1) revealed that EGFL7 (E7) reduced endosomal and lysosomal trafficking of $\alpha_V\beta_3$. Treatment with fibronectin (Fn) alone did not cause significant changes in $\alpha_V\beta_3$ trafficking (n = 11; one-way ANOVA). (**D-F**) Treatment with EGFL7 or Fn reduced intracellular trafficking of integrin $\alpha_5\beta_1$, an effect further enhanced by a combination of both proteins (n = 11; one-way ANOVA). Data presented as mean ± SEM. Scale bars represent 20 µm.

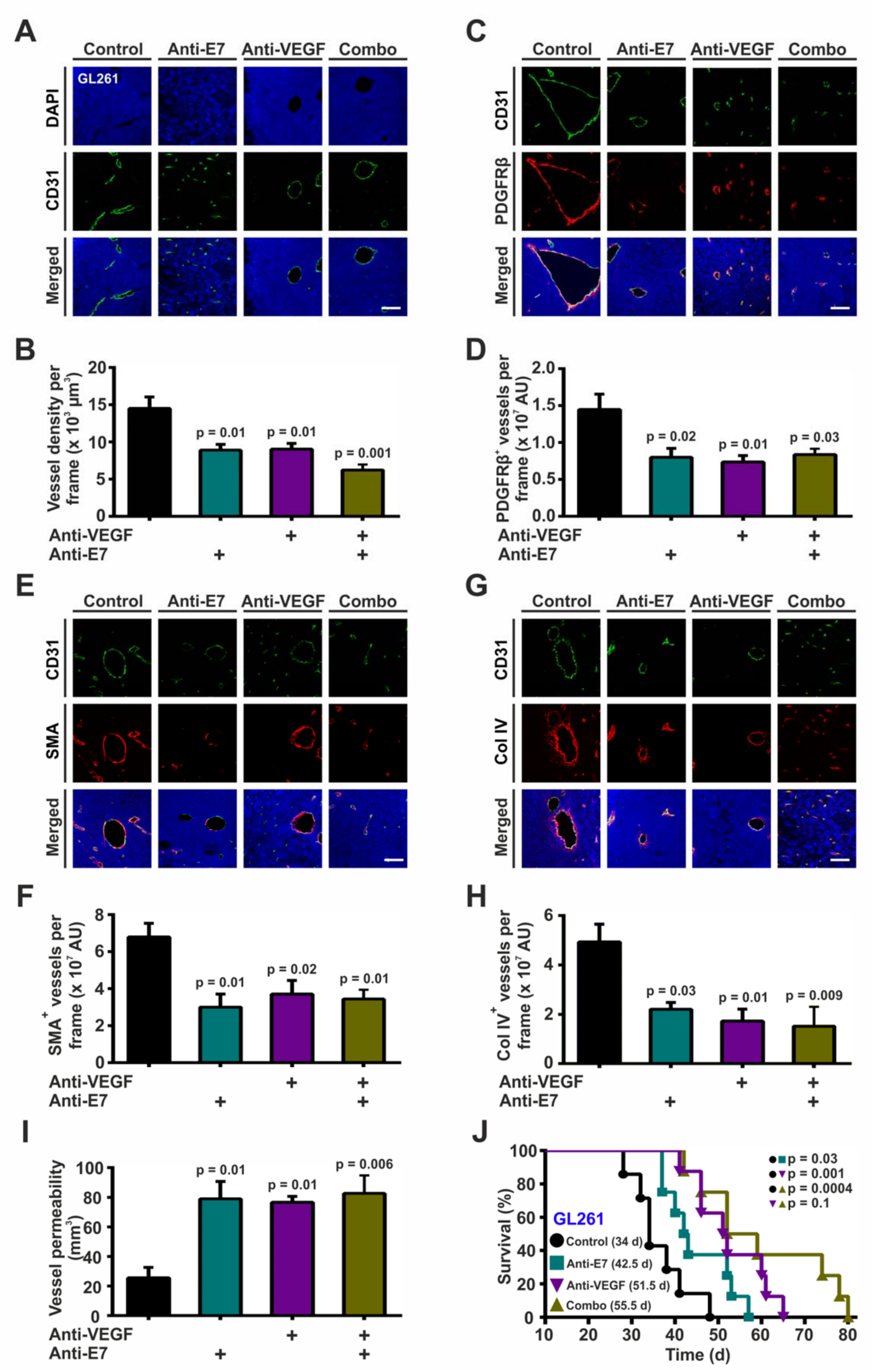
A
Control
Anti-E7
Anti-VEGF
Combo
GL261
DAPI
CD31
Merged
B
Vessel density per frame (x 10³ µm³)
p = 0.01
p = 0.01
p = 0.001
Anti-VEGF
Anti-E7
C
PDGFRβ
D
PDGFRβ⁺ vessels per frame (x 10⁷ AU)
p = 0.02
p = 0.01
p = 0.03
E
SMA
F
SMA⁺ vessels per frame (x 10⁷ AU)
p = 0.01
p = 0.02
p = 0.01
G
Col IV
H
Col IV⁺ vessels per frame (x 10⁷ AU)
p = 0.03
p = 0.01
p = 0.009
I
Vessel permeability (mm³)
p = 0.01
p = 0.01
p = 0.006
J
Survival (%)
Time (d)
GL261
Control (34 d)
Anti-E7 (42.5 d)
Anti-VEGF (51.5 d)
Combo (55.5 d)
p = 0.03
p = 0.001
p = 0.0004
p = 0.1

Figure S6. Anti-EGFL7 treatment for the treatment of glioma *in vivo*

GL261 tumor-bearing mice were injected with anti-EGFL7 or anti-VEGF antibodies, a combination of both (Combo), or isotype negative controls. Mice were sacrificed upon showing first symptoms of disease and brains were analyzed by magnetic resonance imaging (MRI). Resulting brain tumor sections were analyzed for blood vessel density and maturation state by immunohistochemistry. (**A+B**) CD31 staining revealed that blocking of EGFL7, VEGF, or a combination of both lead to decreased tumor vessel density (n = 3; one-way ANOVA). Staining for (**C+D**) PDGFRβ, (**E+F**) SMA, and (**G+H**) Col IV revealed decreased amounts of blood vessel-associated pericytes, smooth muscle cells and Col IV in the basement membrane of blood vessels upon treatment with anti-EGFL7, anti-VEGF and most significantly, a combination of both (n = 3; one-way ANOVA). (**I**) This resulted in an increased intratumoral vessel permeability as measured by T1-weightened MRI analyses of extravasating Gadovist (n = 3; one-way ANOVA). (**J**) Treatment with anti-EGFL7 (42.5 d survival), anti-VEGF (51.5 d), or a combination of both antibodies (55.5 d) increased the median survival time of glioma-bearing animals as compared to isotype-treated control (34 d; n = 7; log-rank test). Data presented as mean ± SEM, AU-arbitrary units. Scale bars represent 60 µm.

Table S1: qRT-PCR primer sequences applied in this study

Gene	Species	Primer	Sequence (5'-3')
EGFL7	Human	hEGFL7_for	TGGATGAATGCAGTGCTAGG
		hEGFL7_rev	CCTTGGGCACACAGAGTGTA
HPRT1	Human	hHPRT1_for	TGAGGATTTGGAAAGGGTGT
		hHPRT1_rev	GAGCACACAGAGGGCTACAA
ACTB	Human	hACTB_for	CATCACCATTGGCAATGAGC
		hACTB_rev	CGATCCACACGGAGTACTTG
EGFL7	Mouse	EGFL7_for	CACCTACCGAACCATCTACC
		EGFL7_rev	ACATGGAGGCTGGCATATTG
GAPDH	Mouse	GAPDH_for	TGAAGCAGGCATCTGAGGG
		GAPDH_rev	CGAAGGTGGAAGAGTGGGAG
RNAPol II	Mouse	RNAPolII_for	GACAAAACTGGCTCCTCTGC
		RNAPolII_rev	GCTTGCCCTCTACATTCTGC
Rps13_3	Mouse	mRPS13_for	TTCACCGATTGGCTCGATAC
		mRPS13_rev	TTATGCCACTAGAGCAGAGG
