# Supplementary figures and images for: EGFL7 enhances surface expression of integrin α5β1 to promote angiogenesis in malignant brain tumors
Source: EMBO Mol Med. 2018 Aug 2;10(9):e8420. doi: 10.15252/emmm.201708420 (PMC6127886; doi:10.15252/emmm.201708420)

**A**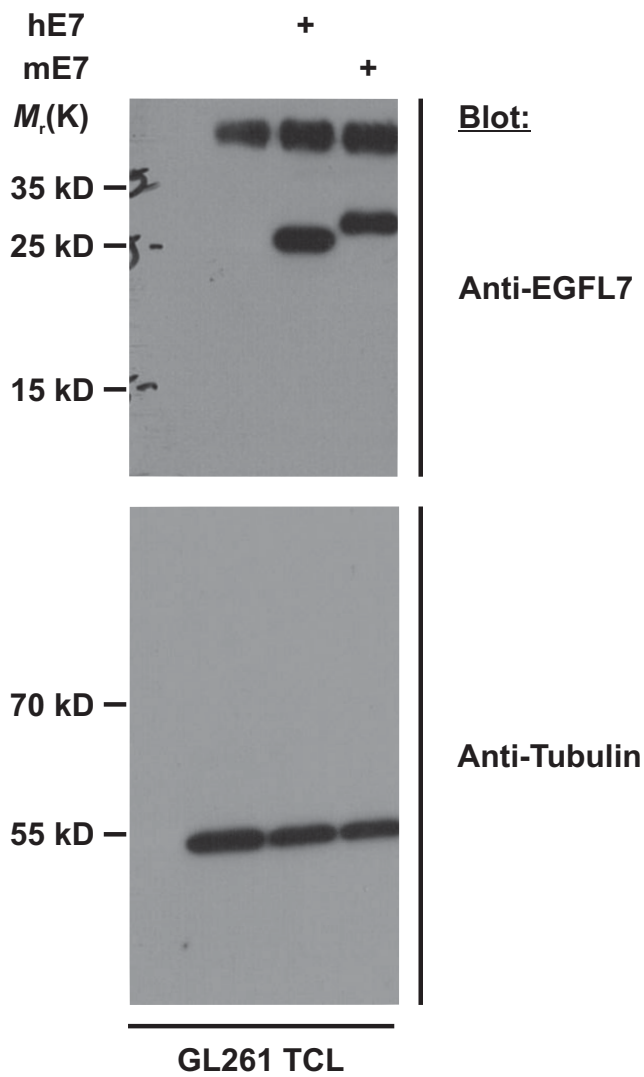**D**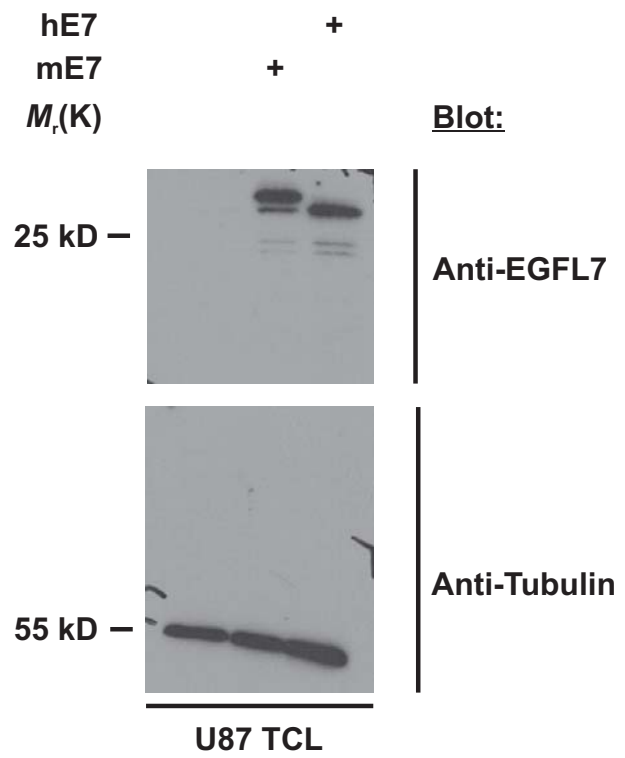**G**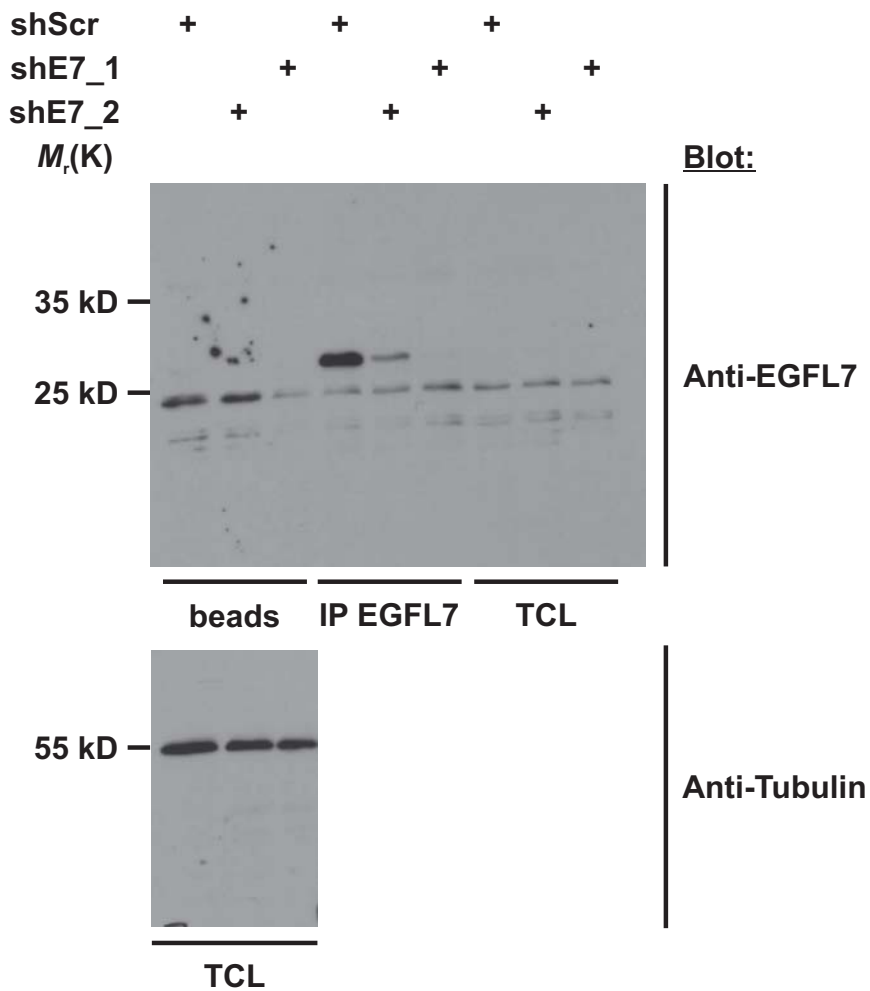

Supplement: Supplementary file 2 — Source Data for Appendix [file EMMM-10-e8420-s004.pdf]

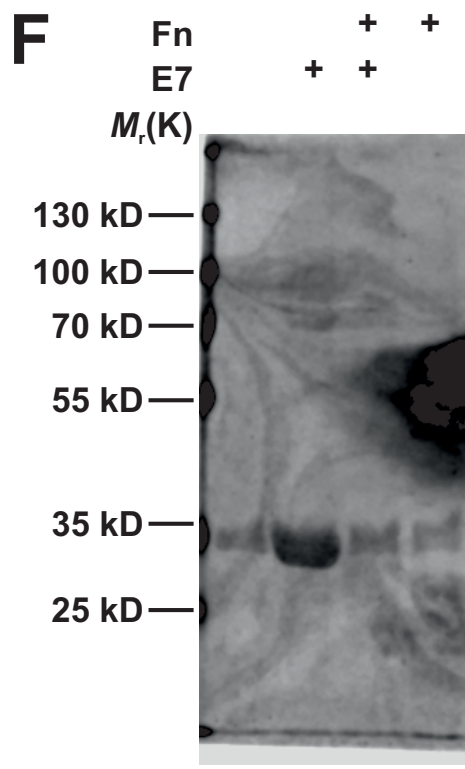

Blot:

Anti-Cdc42

IP Cdc42

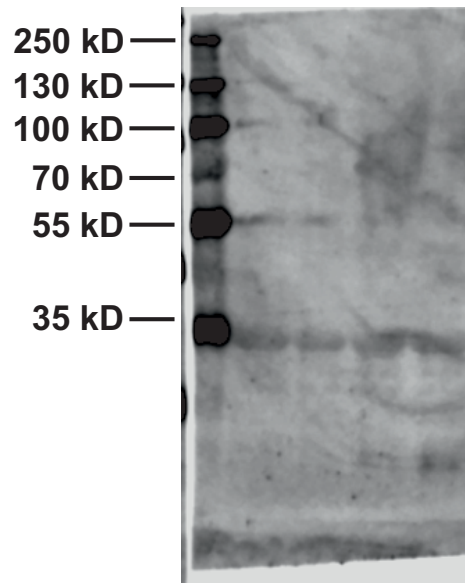

Anti-Rac1

IP Rac1

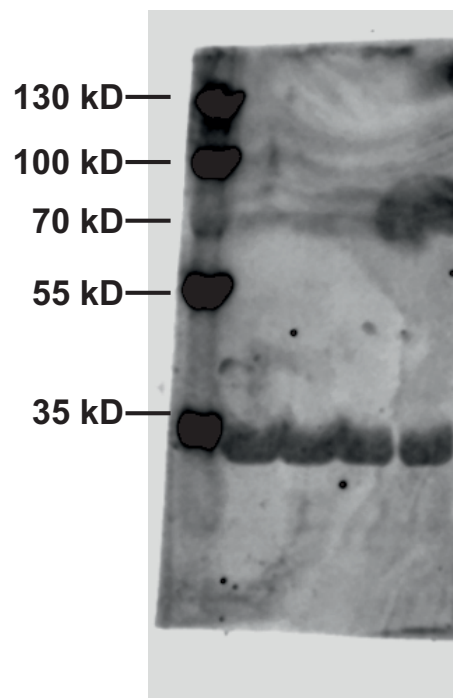

Anti-RhoA

IP RhoA

Supplement: Supplementary file 4 — Source Data for Figure 4 [file EMMM-10-e8420-s002.pdf]

**B**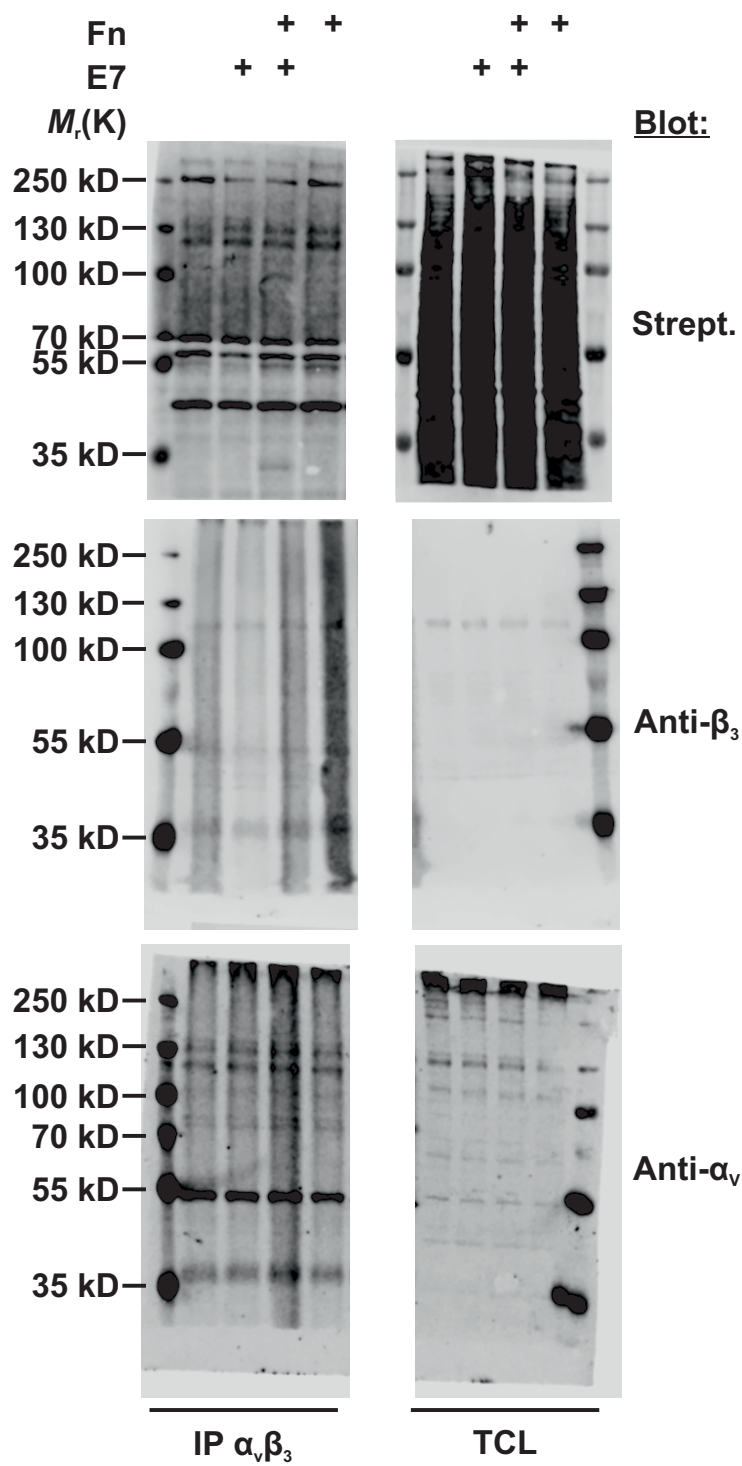**E**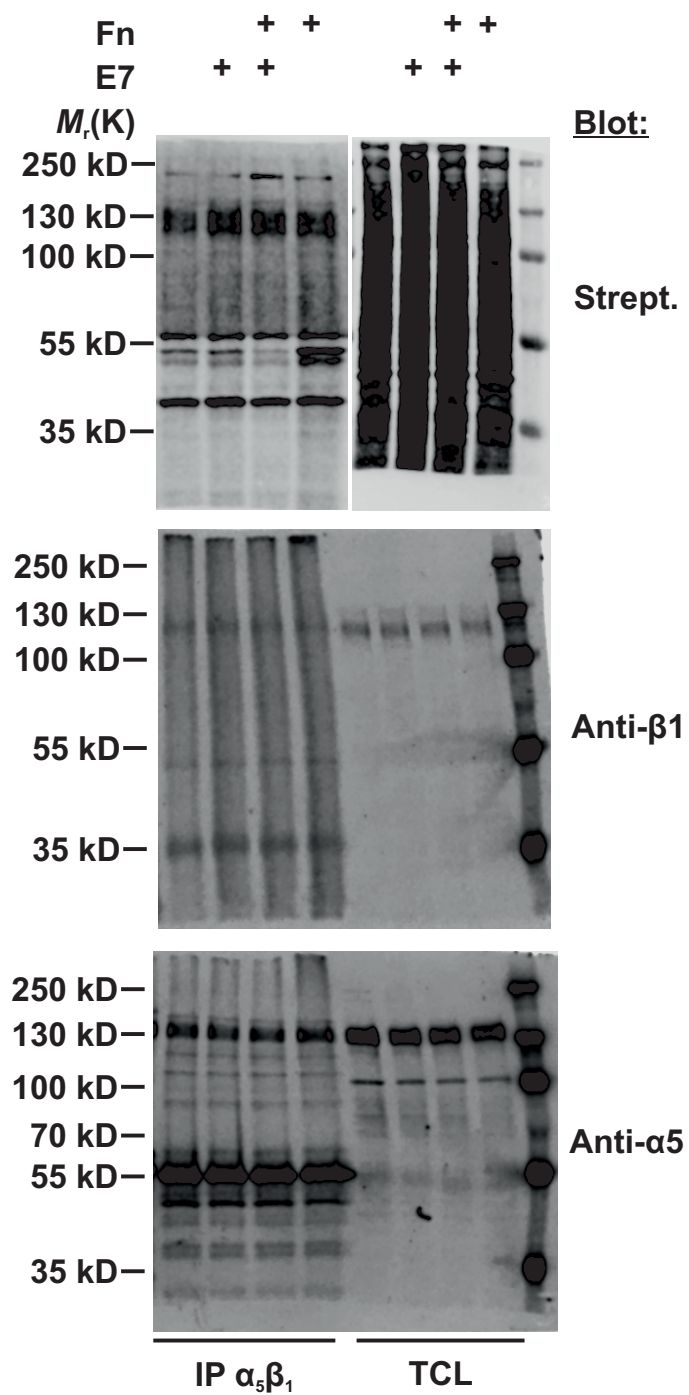

Supplement: Supplementary file 5 — Source Data for Figure 5 [file EMMM-10-e8420-s003.pdf]
